# Flavonoids as a Potential Antifungal Alternative Against *Candida auris* (*Candidozyma auris*) from Clades III and IV

**DOI:** 10.3390/jof12030179

**Published:** 2026-03-02

**Authors:** Jonathan García-Hernández, Omar Gómez-García, Lourdes Villa-Tanaca, Dulce Andrade-Pavón

**Affiliations:** 1 Laboratorio de Biología Molecular de Bacterias y Levaduras, Departamento de Microbiología, Escuela Nacional de Ciencias Biológicas, Instituto Politécnico Nacional, Prol. de Carpio y Plan de Ayala. Col. Sto. Tomás, Mexico City 11340, Mexico; jgarciah1404@alumno.ipn.mx; 2 Departamento de Química Orgánica, Escuela Nacional de Ciencias Biológicas, Instituto Politécnico Nacional, Prol. de Carpio y Plan de Ayala. Col. Sto. Tomás, Mexico City 11340, Mexico; jogomezga@ipn.mx

**Keywords:** flavonoids, *Candida auris*, antifungal, efflux pumps, activity, toxicity

## Abstract

*Candida auris* is a critical emerging pathogen of high priority due to its ability to develop multidrug resistance to various antifungals. Given the increase in cases associated with *C. auris*, it is essential to evaluate new candidates with antifungal potential. In this context, flavonoids represent a promising source for the development of new therapeutic alternatives. In this study eleven flavonoids were evaluated for their antifungal activity against *C. auris* strains from clades III and IV. The flavonoids showed dose-dependent inhibition of *C. auris* growth. Toxicity tests were conducted using the in vivo *Tenebrio molitor* model. The flavonoids exhibited toxicity levels either comparable to or lower than reference antifungals. Also, the study examined the ability of the flavonoids to inhibit efflux pumps. Some of the flavonoids (quercetin, fisetin, hesperetin, luteolin and apigenin) reduced efflux pump activity, which is an important feature since these pumps actively expel antifungal drugs from the cell, reducing the drug’s effectiveness. This suggests that the flavonoids might inhibit efflux pump activity, potentially enhancing the efficacy of antifungal treatments. The study supports the potential of flavonoids as new therapeutic agents for *C. auris*. Since they target efflux pumps, which are a significant mechanism of resistance in *C. auris*, flavonoids could be used either alone or in combination with existing antifungals to improve treatment outcomes.

## 1. Introduction

Fungal infections caused by yeasts belonging to the *Candida* genus are considered a major cause of global incidence, morbidity, and mortality, particularly among immunosuppressed patients [1,2,3]. New *Candida* species have emerged as potential opportunistic pathogens, including *Candida haemulonii* and *Candida (Candidozyma) auris*. *C. auris* was first described and isolated in 2009 from the ear canal of a patient in Japan and has since been recognized as a critical priority pathogen by the World Health Organization [4,5,6,7].

Since its emergence, *C. auris* has been reported in more than 40 countries across six continents, generating concern and uncertainty among healthcare personnel and patient communities worldwide [5,6]. To date, six clades of *C. auris* have been genetically identified. Clade I includes isolates with the highest rates of resistance to fluconazole and amphotericin B. Clade II comprises the isolates most susceptible to antifungal agents and is associated with lower mortality rates, whereas clades I and III exhibit the highest mortality. Clades III and IV contain the most resistant isolates to echinocandins and also display the greatest virulence; notably, clade IV has been linked to the most difficult hospital outbreaks to control. Clade V isolates are typically resistant to fluconazole but remain susceptible to echinocandins and amphotericin B. Finally, clade VI isolates have shown susceptibility to azoles, echinocandins, and pyrimidine analogues [7,8,9,10].

In general, the different clades exhibit distinct susceptibility profiles and resistance mechanisms to the major classes of available antifungals. Among the principal mechanisms involved are mutations in the ERG11 gene and the overexpression of MDR and CDR efflux pumps, the latter group representing a key mechanism underlying azole resistance in many opportunistic fungal pathogens, including *C. auris* [11,12,13].

Given the limited number of available antifungal drugs and the fact that *C. auris* is a multidrug-resistant (MDR) pathogen to the major antifungal classes—including azoles, polyenes, and echinocandins—it is critically important to explore new therapeutic options [14]. In this context, flavonoids have emerged as promising candidates due to their natural abundance and broad spectrum of pharmacological activities, including anticancer, antioxidant, anti-inflammatory, antiviral, antibacterial, and antifungal properties [15,16,17,18,19].

Focusing on the antifungal activity exhibited by flavonoids, studies have shown that each of the major flavonoid families demonstrates inhibitory effects against *Candida* spp., with *C. albicans* being the most extensively investigated species to date [20,21].

In this study we conducted an in vitro evaluation of the effects of 11 flavonoids on the growth of two *C. auris* strains belonging to clades III and IV. We additionally assessed the toxicity of these compounds, evaluated their ability to inhibit efflux pump activity in the same strains. Collectively this work supports the proposal of flavonoids as a therapeutic alternative for the treatment of infections caused by the globally relevant fungal pathogen *C. auris*.

## 2. Materials and Methods

### 2.1. Microorganisms

Two *C. auris* strains were used in this study. *C. auris* strain CJ97 (formerly 49) [22,23], belonging to clade III, was isolated from the bloodstream of a patient with candidemia at Hospital La Fe, Valencia, Spain, and was kindly provided by Dr. Eulogio Valentín-Gómez. *C. auris* strain 20-1498 [24], corresponding to clade IV, was isolated from the bloodstream of a Mexican patient with severe gastrointestinal complications due to endometriosis, hospitalized at San José Hospital, Monterrey, Nuevo León, Mexico, and donated by Dr. Gloria González from the Autonomous University of Nuevo León. *C. albicans* ATCC 10231, *C. krusei* ATCC 6258, and *C. glabrata* CBS138 were included as reference control strains for the efflux pump activity inhibition assays.

### 2.2. Viability, Purity and Conservation of Strains

The viability and purity of the strains were assessed by culturing them on solid YPD (MCD Lab, Tlalnepantla de Baz, Mexico) medium (1% yeast extract, 2% casein peptone, 2% dextrose) at 37 °C for 24 h. The strains were preserved for medium- and long-term storage in 50% glycerol and kept at −70 °C.

The two *Candida auris* strains were differentiated using CHROMagar™ Candida Plus (MCD Lab, Tlalnepantla de Baz, Mexico), according to the manufacturer’s instructions. Strains were streaked onto the medium and incubated at 35–37 °C for 24–48 h. Colony coloration and morphology characteristic of *C. auris* on CHROMagar™ Candida Plus (Fisher Scientific, Waltham, MA, USA) were used for strain differentiation (Supplementary Material Figure S1).

### 2.3. Reference Antifungal Compounds and Flavonoids

**Figure 1:**
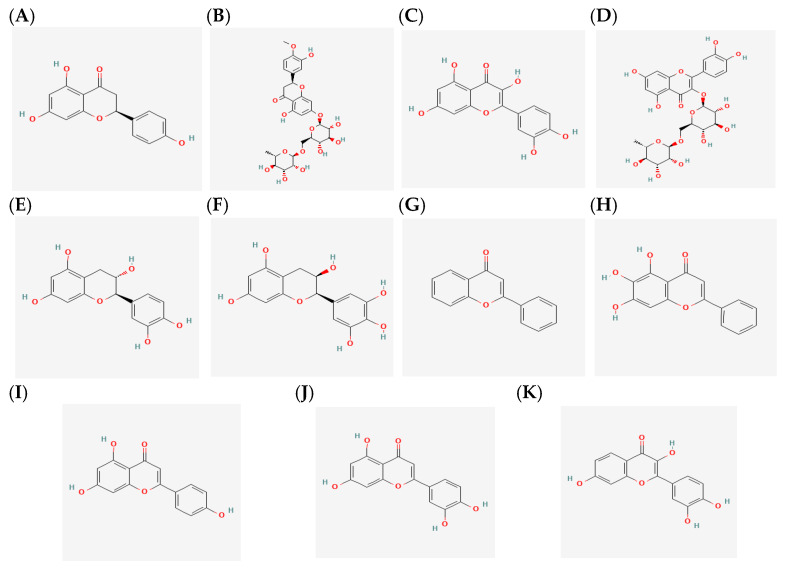
**Flavonoids used in this work.** (**A**) naringenin, (**B**) hesperetin, (**C**) quercetin, (**D**) rutin, (**E**) catechin, (**F**) epigallocatechin, (**G**) flavone, (**H**) baicalein, (**I**) apigenin, (**J**) luteolin and (**K**) fisetin.

Fluconazole, itraconazole, amphotericin B, and caspofungin were used as reference antifungal agents. Eleven flavonoids were evaluated, all purchased from Sigma-Aldrich (St. Louis, MO, USA). These compounds were naringenin, hesperetin, quercetin, rutin, catechin, epigallocatechin, flavone, baicalein, apigenin, luteolin, and fisetin (Figure 1). The selection was based on a literature review aimed at identifying flavonoids previously reported to inhibit *Candida* growth. From this analysis, 11 flavonoids were chosen, prioritizing those most frequently cited in scientific studies while ensuring representation across the main structural families within this class. This selection strategy was designed to facilitate an evaluation of the potential of flavonoids as modulators of antifungal susceptibility and efflux pump activity in *C. auris*.

### 2.4. Evaluation of Flavonoids on Growth Inhibition in C. auris Clades III and IV

*C. auris* strains CJ97 and 20-1498 were precultured for 16 h in YPD medium at 37 °C with shaking at 100 rpm. Exponentially growing cells were inoculated into 96-well microplates containing 300 μL of YPD medium with the indicated treatments, adjusted to an OD_620_ of 0.1 (~1 × 10^6^ cells/mL). Test compounds were added from 10 mM stock solutions to final concentrations of 50–750 μM. YPD medium without compounds and without inoculum served as sterility controls, while YPD medium without compounds served as the growth control. A vehicle control containing final concentration <1% DMSO was included. Background absorbance from wells containing medium and compounds without yeast was subtracted when necessary. Plates were incubated for 24 h at 37 °C with shaking, and OD_620_ was recorded every 30 min using a Multiskan FC microplate reader (Thermo Fisher Scientific, Waltham, MA, USA). Experiments were performed in triplicate, and data were analyzed by two-way ANOVA followed by Dunnett’s post hoc test.

Additionally, colony-forming units (CFUs) for reference antifungals and selected flavonoids were determined. For this purpose, yeast cultures were adjusted to 2–5 × 10^4^ cells/mL and incubated in YPD broth in the presence or absence of each compound at concentrations of 250, 500, and 750 μM. Aliquots were collected at 0, 4, 8, and 12 h of incubation. The samples were then diluted, and 50 μL of each dilution was spread onto YPD agar plates. After incubation for 24 h at 37 °C, colonies were counted to calculate CFU/mL.

### 2.5. Determination of the Minimum Inhibitory Concentrations (MICs) of Flavonoids and Reference Antifungal Compounds Against C. auris Clades III and IV

The effect of eleven flavonoids and four reference antifungal compounds on the growth of two *Candida auris* strains (clades III and IV) was evaluated using the CLSI-standardized M27-A3 broth microdilution method for antifungal susceptibility testing. Yeast strains were cultured for 24 h on YPD agar plates, and isolated colonies were resuspended in sterile saline (0.85% NaCl) and adjusted to 0.5 McFarland units at 620 nm. The inoculum was further diluted 1:1000 in RPMI-1640 medium (Sigma-Aldrich, St. Louis, MO, USA) buffered with MOPS and adjusted to pH 7.0 ± 0.1, which had been sterilized by filtration through a 0.2 μm membrane. Stock solutions of fluconazole and caspofungin were prepared in water, whereas flavonoids, itraconazole, and amphotericin B were dissolved in final concentration <1% DMSO. DMSO (Sigma-Aldrich, St. Louis, MO, USA) and serial dilutions were prepared in RPMI-1640 to obtain final concentrations ranging from 64 to 0.12 μg/mL, with the final DMSO final concentration kept below 1%. Microdilution assays were performed in 96-well plates by adding 100 μL of the adjusted inoculum and 100 μL of each compound concentration, including appropriate sterility, solvent, and growth controls in triplicate. Plates were incubated at 37 °C for 24 and 48 h, and growth was quantified by measuring optical density at 620 nm using a Multiskan FC microplate spectrophotometer (Thermo Fisher Scientific, Waltham, MA, USA). MIC values were defined as the lowest concentration of each compound that completely inhibited visible growth compared with the growth control, while MIC_50_ values were defined as the lowest concentration reducing optical density by 50%.

### 2.6. Evaluation of the Toxicity of Flavonoids Using T. molitor (TM) Larvae as a Model

For this study, *T. molitor* larvae were used as a toxicological model [25]. The larvae, weighing approximately 250 mg, were obtained from the Coatlcalli University Herpetarium at the Benemérita Universidad Autónoma de Puebla (BUAP). Their diet consisted of flaked bran supplemented with slices of fruit or vegetables three times per week. The cultures were maintained at temperatures between 25–28 °C and relative humidity of 50–70%. The acute toxicity assay was initiated by administering an initial dose of each test compound by subcutaneous route (5 mg/kg body weight) to five larvae. Larval mortality was recorded daily. If three or more larvae died, the compound was classified in the highest toxicity category (GHS 1). If three or more larvae survived for five days, the toxicity assessment proceeded by administering the same initial dose (5 mg/kg body weight) to a new cohort of larvae. If three or more larvae from this second cohort survived, a higher dose (50 mg/kg body weight) was administered to five new larvae. The experiment continued in this stepwise manner until a toxic dose was identified. If a compound did not cause mortality at the highest tested dose (2000 mg/kg body weight), it was classified as non-toxic. Isotonic saline solution was used as the non-toxicity control, and final concentration <1% DMSO as the vehicle control.

### 2.7. Measurement of Efflux Pump Inhibition Activity in the Presence and Absence of Flavonoids

The efflux pump activity assay was performed following the protocol described by Ben-Ami et al. (2017) [26], with minor modifications. A preculture was prepared by inoculating 5 mL of *C. auris* 20-1498, *C. auris* CJ97, *C. albicans* ATCC 10231, *C. krusei* ATCC 6258, and *C. glabrata* CBS138 into 95 mL of liquid YPD medium and incubating at 37 °C with orbital shaking at 200 rpm until a cell density of 1 × 10^7^ cells/mL was reached. Yeast cells were harvested by centrifugation at 11,000 rpm for 5 min, resuspended in 20 mL of fresh YPD broth, and incubated at 27 °C for 2 h. The culture was centrifuged again at 11,000 rpm for 5 min and washed twice with PBS, gently vortexing and discarding the supernatant after each wash. The pellets were resuspended in PBS, and 1 mL of each suspension was transferred to 1.5 mL microtubes. To determine the optimal rhodamine 6G (R6G) concentration (Sigma-Aldrich, St. Louis, MO, USA), seven microtubes were prepared per strain: three “positive” and three “negative” tubes containing 15, 30, or 50 μM R6G, along with a control tube without R6G. To evaluate the effect of flavonoids on efflux pump activity, six microtubes were prepared for the *C. auris* strains: three positive and three negative tubes containing flavonoid concentrations of 250, 500, or 750 μM. Glucose 8 mM was added only to the positive tubes. R6G was added at the indicated concentrations from a 1 mM stock solution, adjusting the final volume to 1 mL.

Samples were mixed by gentle pipetting and incubated at 27 °C for 90 min. After incubation, tubes were centrifuged at 13,000 rpm for 5 min, and the supernatant was discarded. Pellets were washed twice with PBS, resuspended in 750 μL of PBS, and supplemented with 250 μL of 8 mM glucose. Microtubes were incubated at 37 °C for 1 h. Finally, samples were centrifuged again at 13,000 rpm for 5 min, and 200 μL of the supernatant were transferred in triplicate into a 96-well microplate. Fluorescence was recorded using a microplate reader at 527 nm (excitation) and 555 nm (emission). Three independent experimental replicates were performed. Standard deviation and statistical significance were assessed by two-way ANOVA using SigmaPlot 12.0 software.

Following the same protocol, synergistic studies of efflux pump activity were conducted in the presence of flavonoids and fluconazole.

### 2.8. Evaluation of Efflux Pump Inhibition by Confocal Microscopy

The two *C. auris* strains were cultured in YD liquid medium at 37 °C with orbital shaking at 200 rpm until logarithmic phase. Cells were then harvested by centrifugation, and 10^7^ cells were inoculated into 10 mL of fresh YD broth and incubated at 27 °C for 2 h. After incubation, cells were collected by centrifugation and washed twice with PBS. The washed pellets were resuspended in 1 mL of PBS supplemented with 15 μM glucose-free R6G, homogenized by gentle vortexing, and incubated at 27 °C for 90 min. Following staining, cells were centrifuged and washed three times with PBS. The pellets were resuspended in microtubes containing 750 μL of PBS, and 250 μL of PBS supplemented with 8 mM glucose was added. At this step, the flavonoids fisetin or catechin (these compounds were selected based on their ability to either decrease or increase efflux pump activity) were added to a final concentration of 500 μM. Control tubes without flavonoids were processed in parallel. All preparations were incubated at 37 °C for 1 h. Cells were washed twice with PBS, and each pellet was resuspended in 1 mL of PBS with gentle vortexing. Aliquots of each preparation were mounted on microscope slides and examined using a confocal microscope with excitation at 514 nm and emission at 560 nm to assess intracellular R6G accumulation.

### 2.9. Molecular Modeling

Three-dimensional homology models corresponding to the *C. auris* ABC and MFS proteins were generated using Modeller version 10.4 [27] and the *C. albicans* pleiotropic multidrug ABC efflux transporter (PDB: 9IUL) as a template. This structure was obtained from the RCSB Protein Data Bank (20 October 2025, http://www.rcsb.org/) based on its high sequence identity to *C. auris* CDR1 (>50%), as determined by BLAST analysis version 2.17.0 (20 October 2025, https://blast.ncbi.nlm.nih.gov/Blast.cgi). Model validation was performed by generating Ramachandran plots using the SAVES 6.0 server (20 October 2025, https://saves.mbi.ucla.edu/). Tertiary structures were visualized with Discovery Studio Client 2020 (20 October 2025, https://www.3ds.com) and further optimized using Molecular Operating Environment (MOE) software, version 2015.10 (20 October 2025, https://www.chemcomp.com/en/Products.htm).

## 3. Results

### 3.1. Evaluation of Growth Inhibition by Flavonoids in C. auris Clades III and IV

**Figure 2:**
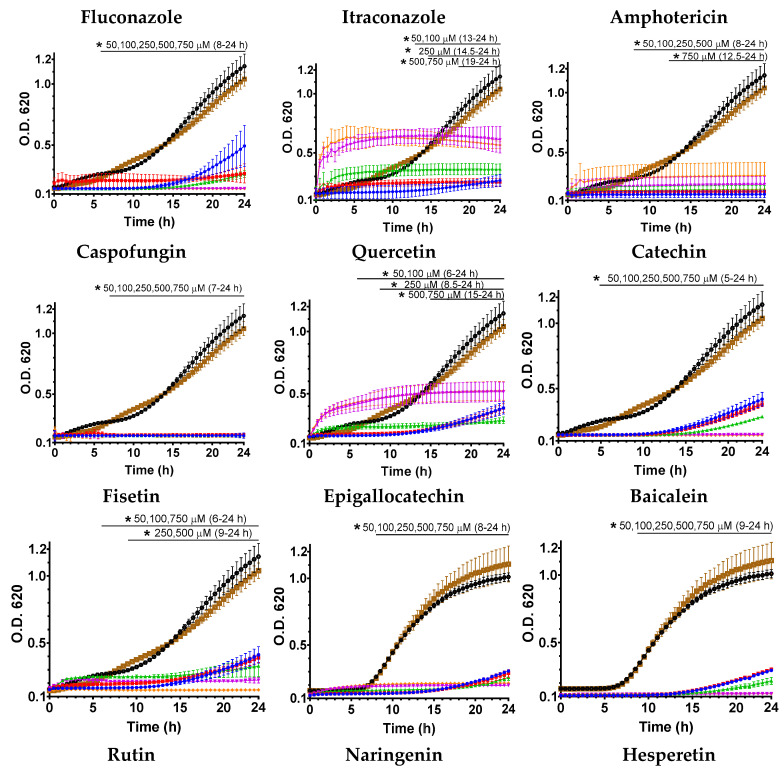

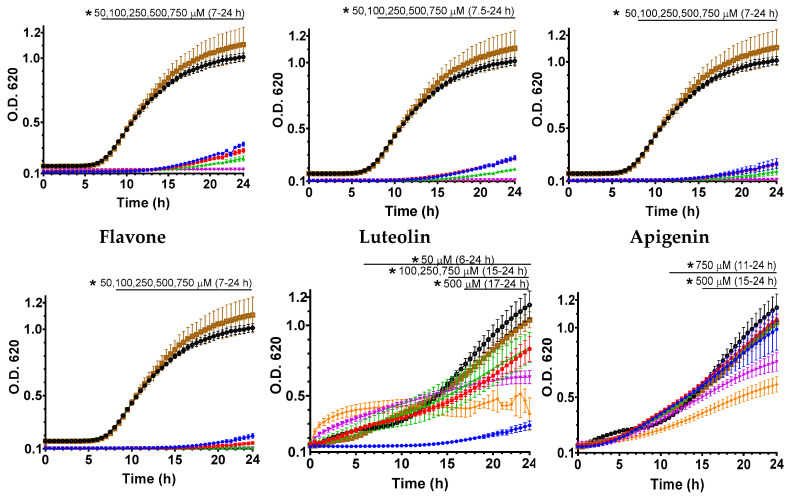
**Effect of flavonoids and reference antifungals on the growth of *C. auris* CJ97 (clade III).** Yeasts were grown in the presence of 50 (blue dots), 100 (red dots), 250 (green dots), 500 (purple dots), and 750 μM (orange dots) as described in the materials and methods section. The controls used were untreated cells (black dotted lines) and cells with DMSO (brown dotted lines). Experiments were performed in triplicate, and the dots represent the mean ± standard error. The horizontal bar indicates the time intervals in which all treatments differed significantly from the untreated (black dots) and DMSO (brown dots) controls (two-way ANOVA, Dunnett’s test, * *p* < 0.05).

**Figure 3:**
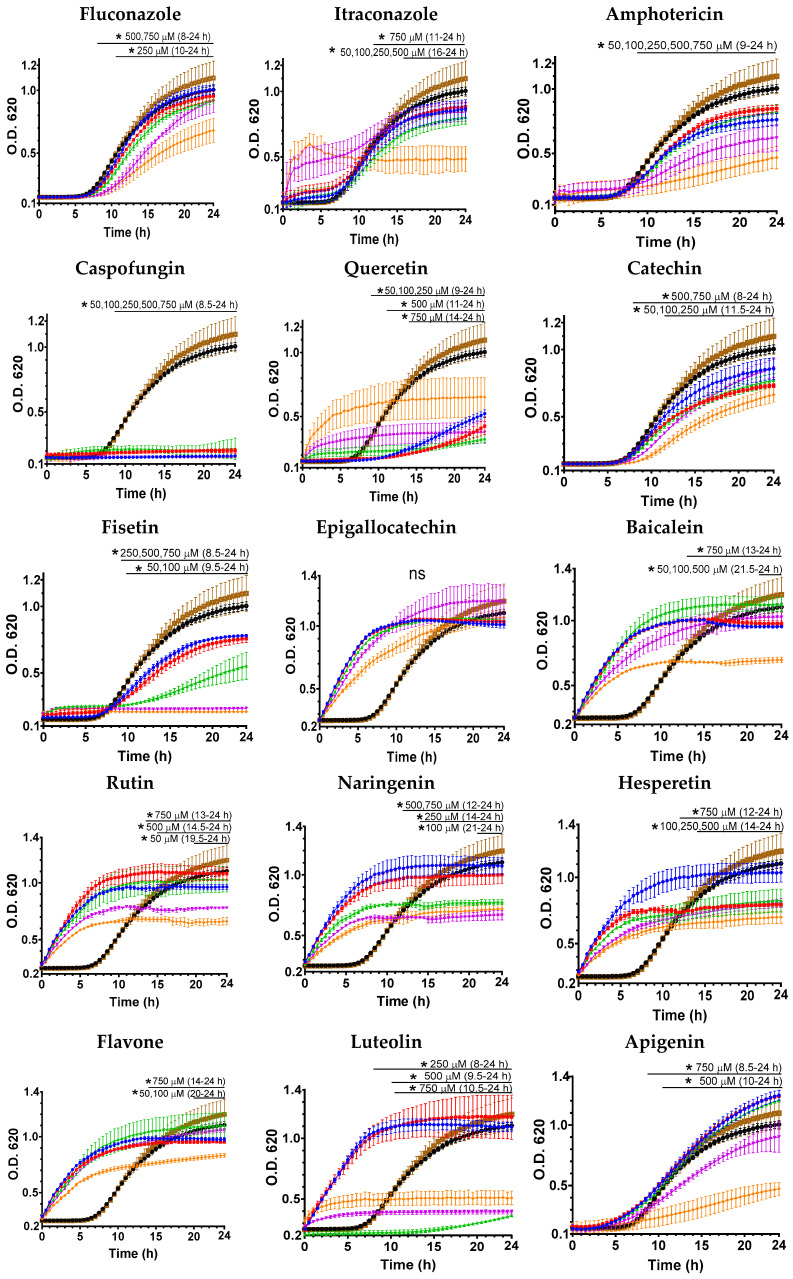
**Effect of flavonoids and reference antifungals on the growth of *C. auris* 20-1498.** Yeasts were grown in the presence of 50 (blue dots), 100 (red dots), 250 (green dots), 500 (purple dots), and 750 μM (orange dots) as described in the materials and methods section. The controls used were untreated cells (black dotted lines) and cells with DMSO (brown dotted lines). Experiments were performed in triplicate, and the dots represent the mean ± standard error. The horizontal bar indicates the time intervals in which all treatments differed significantly from the untreated (black dots) and DMSO (brown dots) controls (two-way ANOVA, Dunnett’s test, * *p* < 0.05, ns = no significant difference).

The effect of flavonoids and reference antifungals on the growth of *C. auris* was evaluated over 24 (Figure 2 and Figure 3) and 48 h (Supplementary Material Figures S2 and S3) using 50, 100, 250, 500, and 750 μM of each compound. Strain CJ97 showed a clear dose-dependent inhibition pattern for both flavonoids and reference antifungals (Figure 2), showing reduced growth relative to the control groups (compound-free medium and DMSO control). This behavior confirmed the strain’s sensitivity to most flavonoids, several of which inhibited growth more effectively at lower concentrations than the two azole antifungals but not amphotericin B or caspofungin. In contrast, quercetin, luteolin, and apigenin required higher concentrations to produce a noticeable inhibition of growth kinetics.

The growth curves strain 20-1498 showed lower sensitivity overall to treatment with both flavonoids and reference antifungals (Figure 3). Several flavonoids—including quercetin, fisetin, baicalein, naringenin, rutin, hesperetin, luteolin, and apigenin inhibited the logarithmic growth phase only at the highest concentrations tested (500–750 μM), displaying a clear dose-dependent effect. A similar pattern was observed for the reference antifungals itraconazole and amphotericin B, which exerted greater inhibition at 750 μM. Among the tested antifungals, only caspofungin inhibited growth at the lowest concentrations (50 μM). In contrast, fluconazole failed to achieve sustained inhibition, and logarithmic growth was observed at all concentrations tested.

In both strains, an apparent loss of dose–response behavior was observed at higher compound concentrations (either flavonoids or reference antifungals) before significant growth had been detected in the controls. Several mechanisms have been documented in *Candida* species, including *C. auris*, that may explain this phenomenon. These include population heterogeneity leading to “rebound” growth curves to the major classes of antifungals (azoles, polyenes, and echinocandins) [28,29,30,31]. These effects may be related to compound aggregation or precipitation at high concentrations, chemical degradation or oxidation, or adaptive yeast responses such as increased efflux pump activity.

To quantify the effect of flavonoids on *C. auris* viability, colony-forming units (CFU) were determined for each treatment and plotted as log CFU/mL over time at the tested concentrations (Supplementary Material Figures S4 and S5). A general dose–response was observed for both antifungals and flavonoids, with higher concentrations leading to progressive growth inhibition. In strain CJ97, fungicidal efficacy was observed only for itraconazole and amphotericin B at the highest concentrations, while fluconazole had no significant effect. Flavonoids reduced CFU, with fungicidal activity requiring 500–750 μM. In strain 20-1498, antifungals had a stronger impact: itraconazole (750 μM) fully inhibited CFU numbers after 8 h, and amphotericin B (500–750 μM) caused complete inhibition from 4 h. Fluconazole showed limited effect, reducing CFU only at 750 μM after 8 h. Flavonoids decreased CFU primarily at the highest concentrations, with hesperetin and luteolin also active at 500 μM, while rutin was ineffective. Overall, flavonoids were mainly fungistatic, whereas most antifungals exhibited fungicidal activity in this strain.

Furthermore, to evaluate the direct effect of flavonoids on the growth of the two *C. auris* strains, the MICs of the eleven flavonoids analyzed, as well as those of the reference antifungal compounds, were determined (Supplementary Material Table S1). The results showed that several flavonoids (rutin, luteolin, hesperetin, and naringenin) exhibited MIC values lower than those observed for fluconazole.

### 3.2. Evaluation of Flavonoid Toxicity in TM Larvae

To directly assess the toxicity of the flavonoids of interest, *TM* larvae were used as an in vivo model [25]. After administering different flavonoid concentrations, larval survival was monitored, and the survival rates of each treated group.

**Figure 4:**
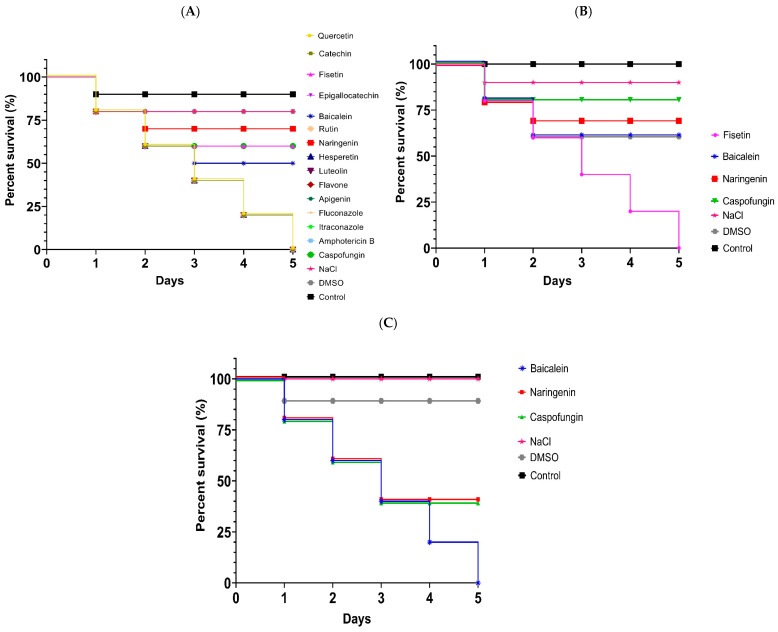
**Survival of *TM* larvae treated with flavonoids and reference antifungals.** Dose of 5 mg/kg (**A**), 50 mg/kg (**B**) and 200 mg/kg (**C**) over a 5-day period were utilized. Control groups included larvae treated with 0.9% NaCl, 10% DMSO, and untreated control.

At the lowest dose administered to the larvae (5 mg/kg), most of the flavonoids tested gave survival rates below 50% (Figure 4A). After 5 days of observation, only the groups treated with fisetin, baicalein, naringenin, and caspofungin exhibited survival rates above 50%. These compounds were therefore selected for further evaluation at the dose level of 50 mg/kg (Figure 4B).

At 50 mg/kg fisetin toxicity survival was below 50% day 3. In contrast, the remaining flavonoids and the reference antifungal caspofungin gave survival rates above 50% throughout the evaluation period. These compounds were therefore advanced to testing at 200 mg/kg.

200 mg/kg baicalein, naringenin, and caspofungin progressively decreased larval survival. After compound administration and five days of monitoring, a progressive decrease with larval survival falling below 50% from the third day.

Of these treatments, baicalein gave the highest level of toxicity, resulting in complete mortality (0% survival) after 5 days. In contrast approximately 40% of larvae treated with naringenin or caspofungin survived.

### 3.3. Measurement of Efflux Pump Inhibition Activity in the Presence and Absence of Flavonoids

The activity of ATP-dependent efflux pumps (ABC family) was evaluated using the chromophore rhodamine 6G (R6G), a widely used substrate for quantifying transporter function. The fluorescence detected in the supernatant after exposure to flavonoid and antifungal treatments allowed assessment of the extent of efflux pump inhibition under the different experimental conditions.

A standard curve was first generated by varying the concentration of R6G, the presence or absence of glucose (8 mM), and the species or strain analyzed. Three R6G concentrations were tested (15, 30, and 50 μM). The addition of glucose induced efflux pump activity, whereas its absence served as a control for reduced transporter function. Figure S6 (Supplementary Material) illustrates the contrast between the two *C. auris* strains examined: 20-1498 (clade IV) and CJ97 (clade III). Strain 20-1498 exhibited higher fluorescence values than CJ97 expressed as relative fluorescence units (RFU), showing the higher transport capacity in strain 20-1498.

Three *Candida* species were evaluated under the same conditions: *C. albicans*, *C. krusei*, and *C. glabrata*. *C. krusei* and *C. glabrata* exhibited higher fluorescence values than those observed for *C. albicans* and *C. auris* CJ97 at 50 μM R6G. However, strain 20-1498 demonstrated the highest efflux activity (Figure S7, Supplementary Material).

**Figure 5:**
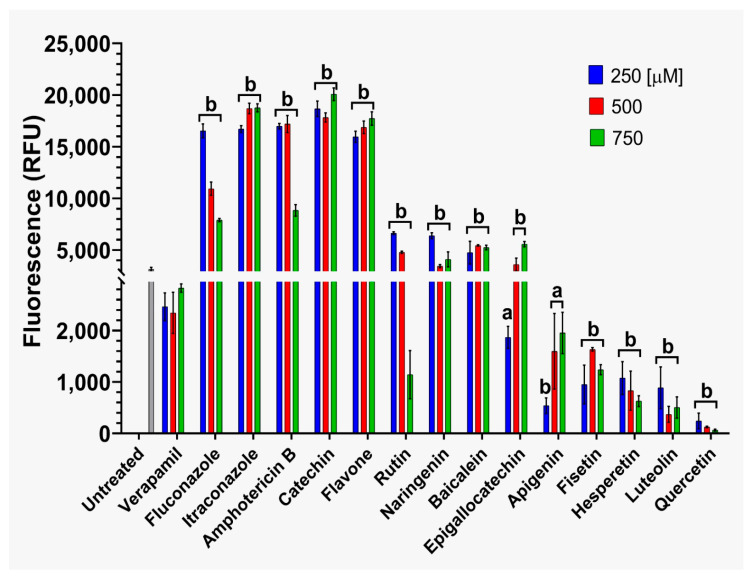
**Assessment of ATP-dependent efflux pump (ABC) activity in *C. auris* CJ97.** The compounds were tested at concentrations of 250, 500, and 750 μM. The dashed line on the scale was included to enhance visualization of the results. Experiments were performed in triplicate and analyzed using two-way ANOVA followed by Dunnett’s post hoc test. Letter **a** indicates *p* > 0.05 versus the verapamil control, whereas letter **b** indicates *p* < 0.05 versus the verapamil control.

**Figure 6:**
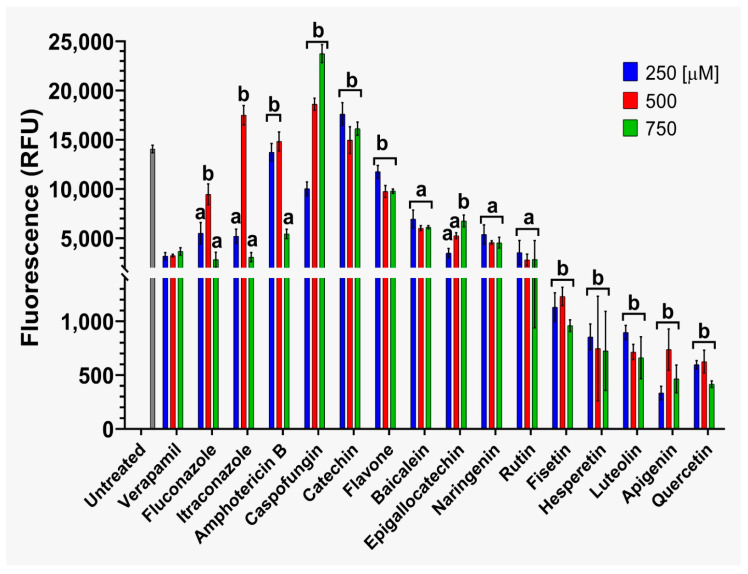
**Assessment of ATP-dependent efflux pump (ABC) activity in *C. auris* 20-1498.** The compounds were tested at concentrations of 250, 500, and 750 μM. The dashed line on the scale was included to enhance visualization of the results. Experiments were performed in triplicate and analyzed using two-way ANOVA followed by Dunnett’s post hoc test. Letter **a** indicates *p* > 0.05 versus the verapamil control, whereas letter **b** indicates *p* < 0.05 versus the verapamil control.

The effects on ABC efflux pump activity by flavonoids and reference antifungals in strains CJ97 (Figure 5) and 20-1498 (Figure 6) were evaluated at three concentrations (250, 500, and 750 μM). In strain CJ97 (Figure 5), treatments with quercetin, fisetin, hesperetin, luteolin, and apigenin at 250 μM exhibited fluorescence values lower than verapamil control, suggesting that these compounds inhibit efflux activity. In contrast, catechin, flavone, and all tested antifungal agents produced higher fluorescence levels, compared with controls indicating efflux pump activation.

For strain 20-1498, five flavonoids (quercetin, fisetin, hesperetin, luteolin, and apigenin) produced fluorescence values lower than the verapamil control, suggesting reduced R6G efflux and, consequently, inhibition of ABC transporter activity. In contrast, catechin and flavone exhibited elevated fluorescence values, indicating a possible increase in pump activity. The reference antifungals (fluconazole, itraconazole, caspofungin, and amphotericin B) increased efflux pump activity.

The increased activity of ABC and MFS transporters observed in the presence of conventional antifungals can be attributed to the activation of cellular defense mechanisms in *C. auris*. Fluconazole is a well-recognized substrate of these efflux pumps, and its exposure directly induces their activity to reduce intracellular drug accumulation. In contrast, although amphotericin B and caspofungin are not considered classical substrates of ABC or MFS transporters, the membrane or cell wall damage they cause may trigger general stress responses, including the indirect activation of efflux transporters as a survival mechanism.

In summary findings suggest that the flavonoids fisetin, hesperetin, apigen and quercetin appear to be inhibitors of ABC transporter-mediated efflux of R6G while catechin and flavone activate efflux of this substrate.

On the other hand, co-treatment of *C. auris* strains CJ97 and 20-1498 (Supplementary Material Figures S8 and S9) with flavonoids and fluconazole led to a marked decrease in energy-dependent efflux pump activity compared with the verapamil control. In both strains, the strongest effects were observed with quercetin, fisetin, epigallocatechin, apigenin, and luteolin, which promoted enhanced intracellular retention of R6G. These results indicate that combining flavonoids with fluconazole produces additive effects, effectively reducing the activity of energy-dependent efflux mechanisms in *C. auris*.

### 3.4. Assessment of Efflux Pump Activity in C. auris CJ97 and 20-1498 Using Confocal Microscopy

**Figure 7:**
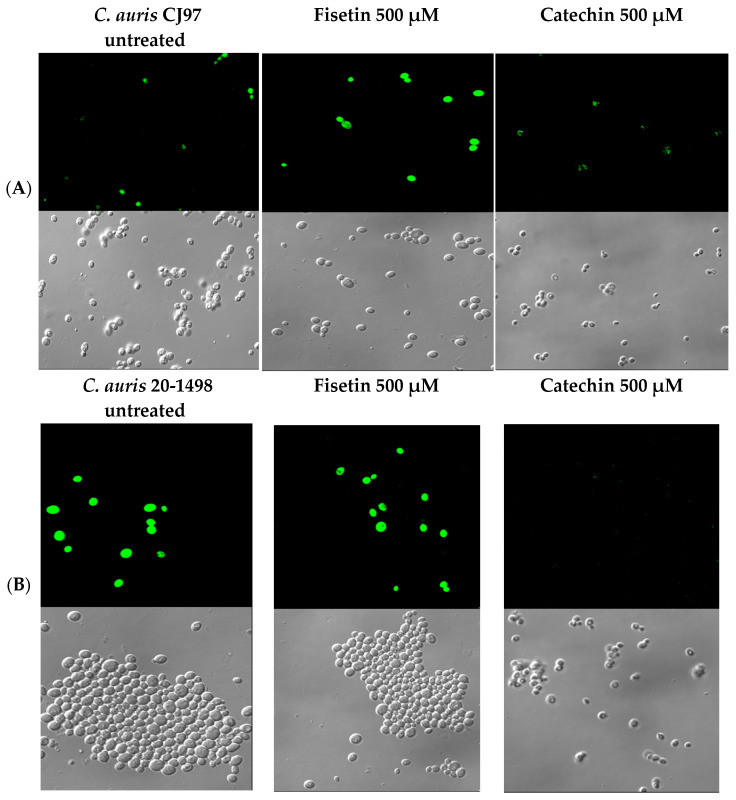
**Activation and inhibition of drug efflux by selected flavonoids in *C. auris* CJ97 and 20-1498 detected by confocal microscopy.** Fluorescent field (**A**) and bright-field images (**B**) are shown.

Confocal microscopy analyses were performed on both *C. auris* strains treated with fisetin or catechin (flavonoids selected based on their capacity to inhibit or increase efflux pump activity, respectively) in the presence of 15 μM R6G and 8 mM glucose. As shown in Figure 7A,B, the untreated CJ97 and 20-1498 strains exhibited intracellular accumulation of R6G, indicating active efflux pump function. In contrast, catechin-treated cells showed very weak fluorescence signals, comparable to or lower than those observed in untreated controls, indicating minimal intracellular R6G retention and limited inhibition of efflux pump activity. Importantly, the qualitative increase in intracellular R6G fluorescence observed upon fisetin treatment was consistently reproduced across multiple independent fields and biological replicates, supporting the conclusion that fisetin impairs R6G extrusion and inhibits ABC transporter-mediated efflux.

Fisetin treatment resulted in a marked increase in intracellular fluorescence, with a greater number of labeled cells and higher signal intensity, consistent with the inhibition of efflux pump activity observed in the R6G efflux assays. Conversely, catechin-treated cells showed minimal fluorescence, reflecting low intracellular accumulation of R6G and indicating little to no inhibition of efflux activity.

### 3.5. Computational Modeling of Multidrug Efflux Transporters from C. auris (Clades III and IV)

Three-dimensional (3D) structures of the ABC and MFS transporters of *C. auris* from clades III and IV were generated. For ABC transporters, the *C. albicans* multidrug ABC efflux transporter Cdr1 (PDB: 9IUL) [32] and the model available in the SWISS-MODEL database were used as templates. For the MFS transporters, the template corresponded to a putative transmembrane transporter of the major facilitator superfamily from *C. intermedia* obtained from the AlphaFold Protein Structure Database version 2.0 (20 October 2025, https://alphafold.ebi.ac.uk) [33]. Homology models were constructed using the Modeller version 10.4 program (20 October 2025, https://salilab.org/modeller/) [27] to generate fifteen models for each transporter with the best model in each case selected based on structural alignment quality.

**Figure 8:**
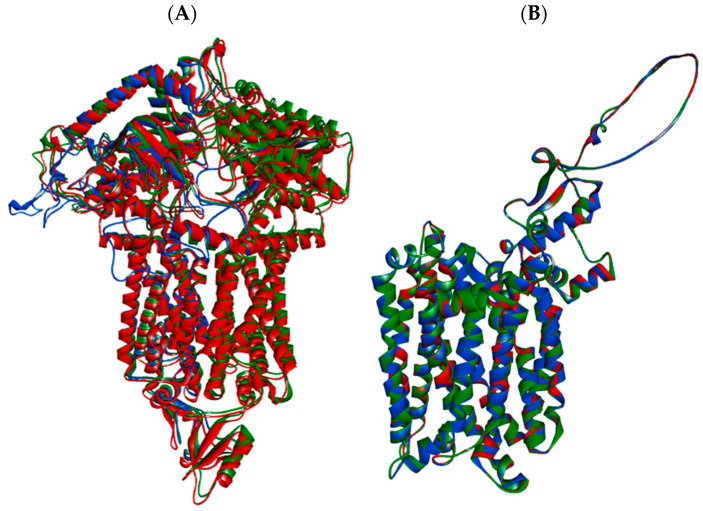
**Overlap of the 3D models of the ABC (A) and MFS (B) transporters from *C. auris* clades III (blue) and IV (red).** The template used is highlighted in green.

The finalized 3D models for ABC and MFS transporters are shown in Figure 8A,B. The ABC transporters from clades III and IV displayed 72.7% and 73.6% primary sequence identity to the template, respectively, and 99.1% identity to each other. Similarly, the MFS transporters from both clades shared 99.4% identity, and 71.8% and 71.6% identity with their corresponding templates. These results indicate that the efflux pumps of clades III and IV have highly conserved structures and conformations. Consequently, the inhibitors evaluated in this study are expected to recognize and interact with the efflux transporters across clades in comparable manners.

To assess the stereochemical quality of the generated models, Ramachandran plots were constructed to analyze the three-dimensional conformation of the proteins based on the distribution of their φ and ψ dihedral angles, derived from their primary structure (Figure S10, Supplementary Material). The analysis showed for each model that more than 90% of the amino acid residues were located within allowed regions. This indicates the structural quality of the modeled proteins and supports their reliability for subsequent computational and functional studies.

## 4. Discussion

Fungal infections are a serious global health threat, causing over 1.5 million deaths annually [3]. This threat is exacerbated by the emergence of multidrug-resistant strains. *C. auris*, which spread rapidly in healthcare settings, persists on surfaces, and is difficult to eradicate. Clinically, a highly significant feature is its intrinsic multidrug resistance [34,35]. The search for new antifungal therapies is critical. Flavonoids exhibit promising antifungal properties, including inhibition of growth, biofilm formation, and efflux pump activity [36,37]. While mostly studied in *C. albicans*, their effects on *C. auris* remain largely unexplored, particularly regarding efflux pump inhibition, a key mechanism of multidrug resistance [6]. In this study, *C. auris* clade III and IV strains were selected for their clinical relevance and high ABC and MFS efflux pump activity. Clades with robust and reproducible efflux phenotypes were prioritized to allow a sensitive and reliable evaluation of the modulatory effects of 11 flavonoids across genetically distinct lineages.

Growth kinetics of *C. auris* in the presence of flavonoids showed variable antifungal effects. In strain CJ97, most flavonoids inhibited growth even at low concentrations (50 μM), except quercetin, luteolin, and apigenin. Strain 20-1498 was more resistant, with only quercetin, catechin, and fisetin effective, while inhibition for other compounds occurred mainly at 500–750 μM. Some treatments accelerated early growth, suggesting a hormesis effect, where low concentrations stimulate growth while high concentrations inhibit it [38,39]. Subinhibitory flavonoid or antifungal concentrations have also been reported to increase cell adhesion and biofilm formation, reflecting the complexity of fungal responses [37,40].

An acute in vivo toxicity assessment of flavonoids and antifungals was performed using *T. molitor* larvae [25]. *T. molitor* has emerged as a useful and reproducible in vivo system for studying fungal infections, host–pathogen interactions, and antifungal efficacy [25]. This model offers practical advantages such as ease of handling, low cost, and ethical acceptability. Therefore, *T. molitor* represents a valuable preliminary model for assessing antifungal toxicity and efficacy prior to validation in mammalian systems. Some flavonoids showed toxicity at low doses; results were comparable to most reference antifungals, suggesting a wide safety margin for baicalein and naringenin. These findings provide initial preclinical evidence to identify compounds requiring further evaluation before pharmacological use [41,42,43,44]. The high larval mortality observed after treatment with conventional antifungal agents in *T. molitor* can be attributed to the nonspecific toxicity of these compounds in invertebrate models, which has been widely documented in the literature. Antifungals such as fluconazole, amphotericin B, and caspofungin have previously been reported to induce systemic stress, metabolic disturbances, and tissue damage in insect models, which explains the high mortality rates observed and supports the use of *T. molitor* as a sensitive model for evaluating toxicity and fungal virulence [25,42]. In contrast, studies have demonstrated that flavonoids and plant extracts exhibit low toxicity in vivo and in invertebrate models, respectively [21,43]. In addition, it has been widely reported that many of these flavonoids are capable of modulating the activity of ABC and MFS efflux transporters, acting as inhibitors or resistance modulators, thereby supporting the hypothesis proposed in this study [45,46].

Efflux pump activity, a key determinant of multidrug resistance, was assessed using a fluorometrically quantified R6G efflux assay. Among the species tested, *C. auris* strain 20-1498 exhibited the highest efflux activity, surpassing both strain CJ97 and the other species evaluated. The observed heterogeneity between the two *C. auris* strains may reflect clade-specific differences in the regulation and genomic composition of efflux-associated genes [46,47,48].

In *C. auris* strains CJ97 and 20-1498 quercetin, fisetin, epigallocatechin, apigenin, and luteolin significantly reduced R6G efflux, demonstrating their capacity to interfere with ABC transporter function. These effects likely result from a combination of mechanisms, including partial blockade of substrate-binding sites, disruption of membrane potential, apoptosis and decreased ATP availability due to mitochondrial impairment [49,50,51,52,53,54,55,56,57]. When combined with fluconazole, the same flavonoids produced an additive effect, further reducing energy-dependent efflux activity and enhancing intracellular retention of R6G in both strains compared with the verapamil control. This suggests that co-treatment can more effectively attenuate efflux-mediated drug resistance, likely through sustained transporter inhibition, interference with proton gradients, and limitation of ATP-driven active efflux. Together, these results highlight the potential of flavonoids, alone or in combination with conventional antifungals, as modulators of efflux pump activity and as promising adjuncts in the management of multidrug-resistant *C. auris* infections. In *C. auris*, flavonoids can affect plasma membrane organization, redox homeostasis, and mitochondrial function, thereby influencing antifungal uptake, oxidative stress responses, and cellular viability [58,59,60]. They have also been shown to modulate signaling pathways and gene expression associated with stress responses and antifungal resistance [61,62]. Additionally, confocal microscopy confirmed that fisetin, but not catechin, increased intracellular R6G retention, validating its inhibition of ABC transporters in *C. auris*.

In general, the flavonoids evaluated in this study have been widely reported to possess antifungal, antioxidant, and drug resistance-modulating activities across various fungal species. In this context, the novelty of the present study lies in providing evidence that flavonoids may contribute to reversing the main efflux-mediated antifungal resistance mechanism through inhibition of ABC and MFS transporter activity in the multidrug-resistant yeast *C. auris*.

## 5. Conclusions

In conclusion, the findings presented in this study have revealed that flavonoids in two strains of *C. auris* exhibited growth inhibition, toxicity comparable to or better than some reference antifungals and inhibition of efflux pump activity. This translates into a potential avenue for reducing *C. auris* resistance. In this way, they could allow existing antifungal agents to retain their efficacy, which is crucial for treating infections that would otherwise be difficult to control. This approach not only promises to improve treatments but could also lead to a better understanding of how to combat resistance mechanisms in various fungal species. Future studies will be essential to confirm the efficacy of flavonoids in clinical practice, but this study represents a step forward in the fight against fungal infections caused by multidrug-resistant yeasts.

## Data Availability

The original contributions presented in this study are included in the article/Supplementary Material. Further inquiries can be directed to the corresponding authors.

## Supplementary Materials

The following supporting information can be downloaded at: https://www.mdpi.com/article/10.3390/jof12030179/s1. Figure S1. Chromogenic differentiation and species-level confirmation of *C. auris* isolates on CHROMagar™ Candida Plus. Figure S2. Effect of flavonoids and reference antifungals on the growth of *C. auris* CJ97 (clade III). Figure S3. Effect of flavonoids and reference antifungals on the growth of *C. auris* 20-1498. Figure S4. Effects of flavonoids and reference antifungal agents on the viability of *C. auris* CJ97 (clade III). Figure S5. Effects of flavonoids and reference antifungal agents on the viability of *C. auris* 20-1498 (clade IV). Figure S6. Assessment of ATP-dependent (ABC-type) efflux pump activity in *C. auris* strains 20-1498 and CJ97. Figure S7. Evaluation of ATP-dependent efflux pump (ABC transporter) activity in *C. auris* strains 20-1498 and CJ97, and in *C. albicans*, *C. krusei*, and *C. glabrata*. Figure S8. Assessment of ATP-dependent efflux pump (ABC transporter) activity in *C. auris* CJ97. Figure S9. Assessment of ATP-dependent efflux pump (ABC transporter) activity in *C. auris* 20-1498. Figure S10. Evaluation of the 3D models of the efflux pumps of *C. auris* from clades III and IV. Table S1. Minimum inhibitory concentration (MIC) results of flavonoids and reference compounds against *C. auris* (clades III and IV). Table S2. MIC values converted from μg/mL to micromolar concentrations (μm). Table S3. Tested concentrations expressed as multiples of the MIC (×MIC) in *C. auris* CJ97. Table S4. Tested concentrations expressed as multiples of the MIC (×MIC) in *C. auris* 201498.
