# Supplementary material for: Flavonoids as a Potential Antifungal Alternative Against Candida auris (Candidozyma auris) from Clades III and IV
Source: J Fungi (Basel). 2026 Mar 2;12(3):179. doi: 10.3390/jof12030179 (PMC13028025; doi:10.3390/jof12030179)
Supplement: Supplementary file 1 [file jof-12-00179-s001.zip › jof-4032937-supplementary.pdf]

Supplementary Material

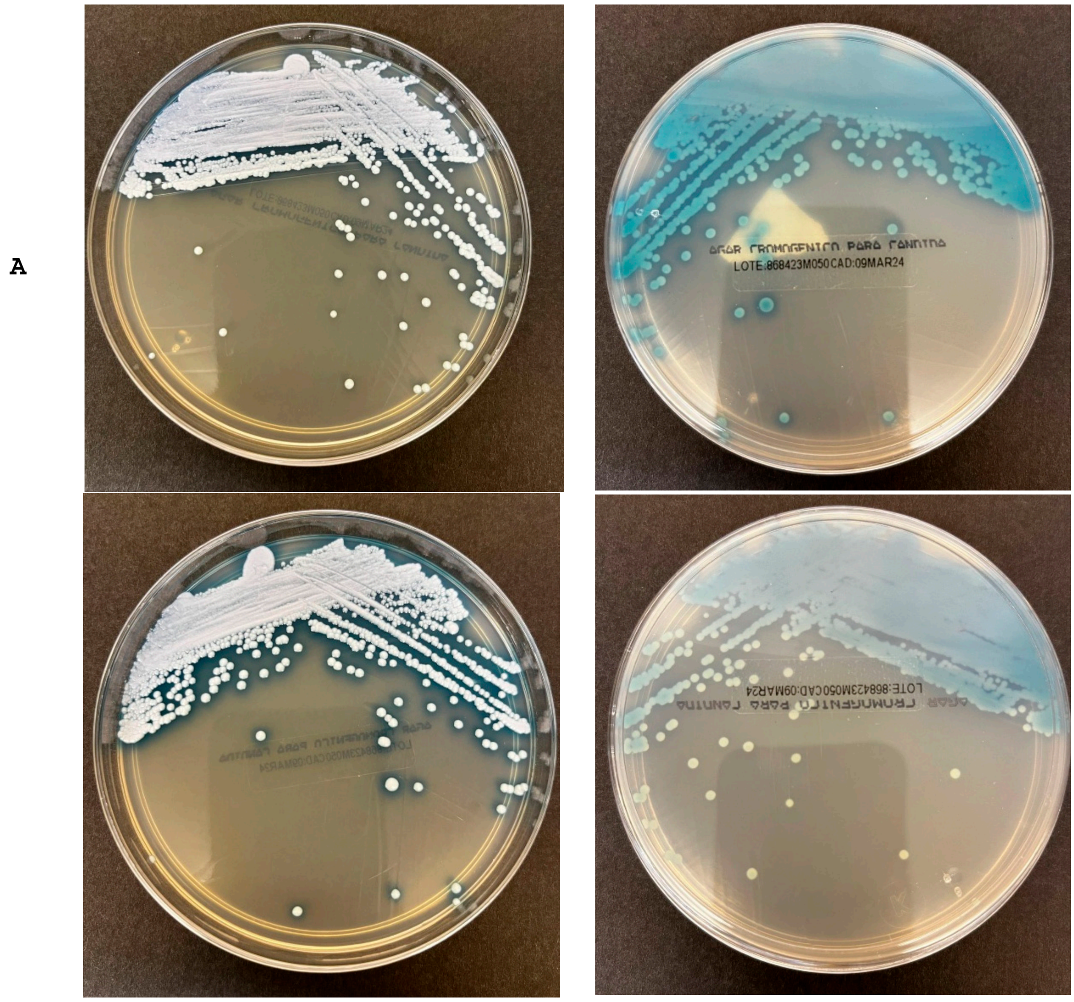

Figure S1. Chromogenic differentiation and species-level confirmation of *C. auris* isolates on CHROMagar™ Candida Plus. (A) Growth of *C. auris* strain CJ97, showing the characteristic colony coloration and morphology that distinguish this species from other *Candida* spp. (B) Growth of *C. auris* strain 20-1498 under identical conditions, exhibiting the same chromogenic phenotype, thereby confirming species identity.

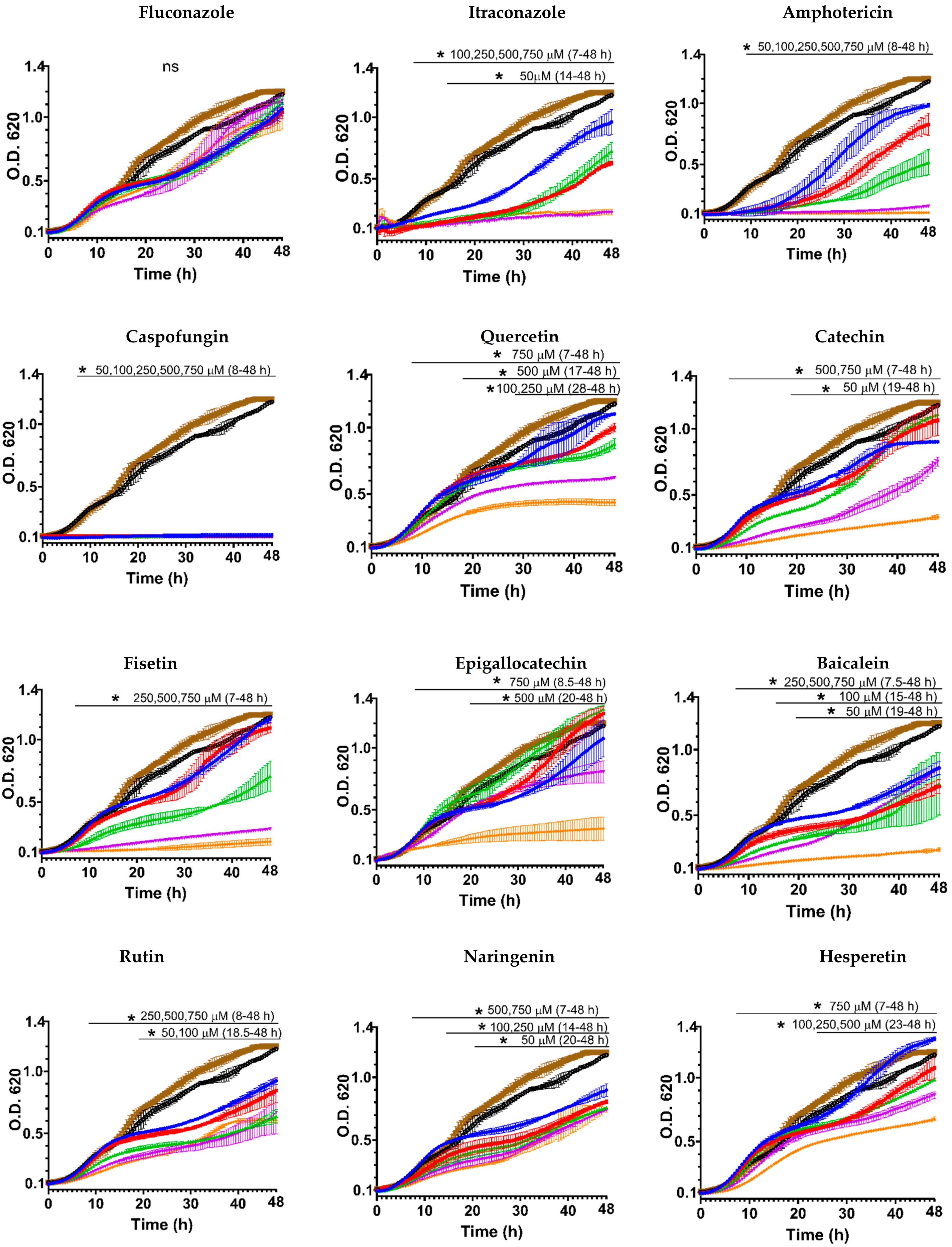

Fluconazole
ns
Itraconazole
* 100,250,500,750 μM (7-48 h)
* 50μM (14-48 h)
Amphotericin
* 50,100,250,500,750 μM (8-48 h)
Caspofungin
* 50,100,250,500,750 μM (8-48 h)
Quercetin
* 750 μM (7-48 h)
* 500 μM (17-48 h)
*100,250 μM (28-48 h)
Catechin
* 500,750 μM (7-48 h)
* 50 μM (19-48 h)
Fisetin
* 250,500,750 μM (7-48 h)
Epigallocatechin
* 750 μM (8.5-48 h)
* 500 μM (20-48 h)
Baicalein
* 250,500,750 μM (7.5-48 h)
* 100 μM (15-48 h)
* 50 μM (19-48 h)
Rutin
* 250,500,750 μM (8-48 h)
* 50,100 μM (18.5-48 h)
Naringenin
* 500,750 μM (7-48 h)
* 100,250 μM (14-48 h)
* 50 μM (20-48 h)
Hesperetin
* 750 μM (7-48 h)
* 100,250,500 μM (23-48 h)
O.D. 620
Time (h)

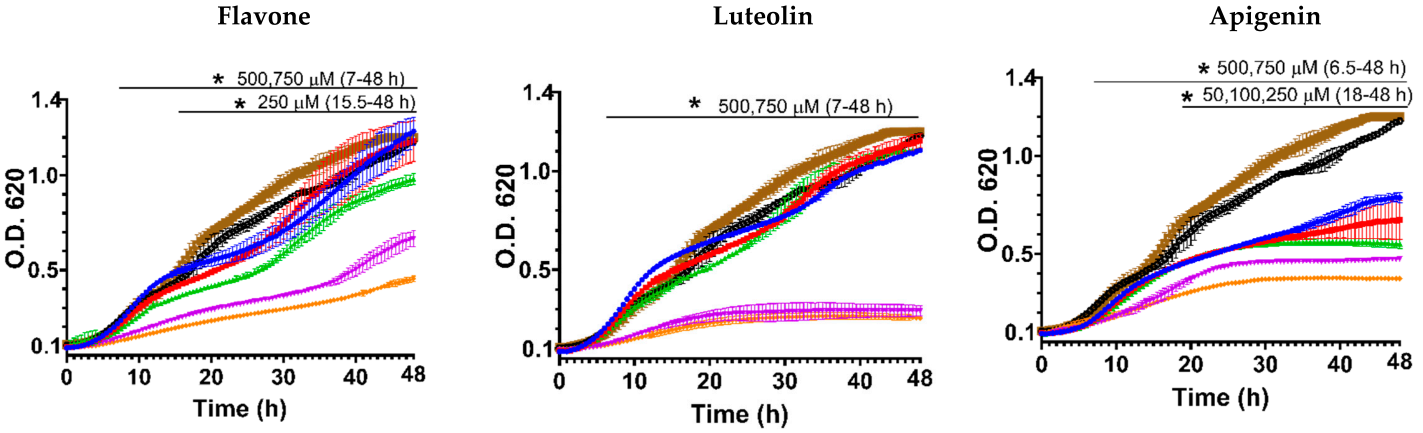

Figure S2. Effect of flavonoids and reference antifungals on the growth of *C. auris* CJ97 (clade III). Yeasts were grown in the presence of 50 (blue dots), 100 (red dots), 250 (green dots), 500 (purple dots), and 750 µM (orange dots) as described in the materials and methods section. The controls used were untreated cells (black dotted lines) and cells with DMSO (brown dotted lines). Experiments were performed in triplicate, and the dots represent the mean ± standard error. The horizontal bar indicates the time intervals in which all treatments differed significantly from the untreated (black dots) and DMSO (brown dots) controls (two-way ANOVA, Dunnett's test, * $p < 0.05$, ns = no significant difference).

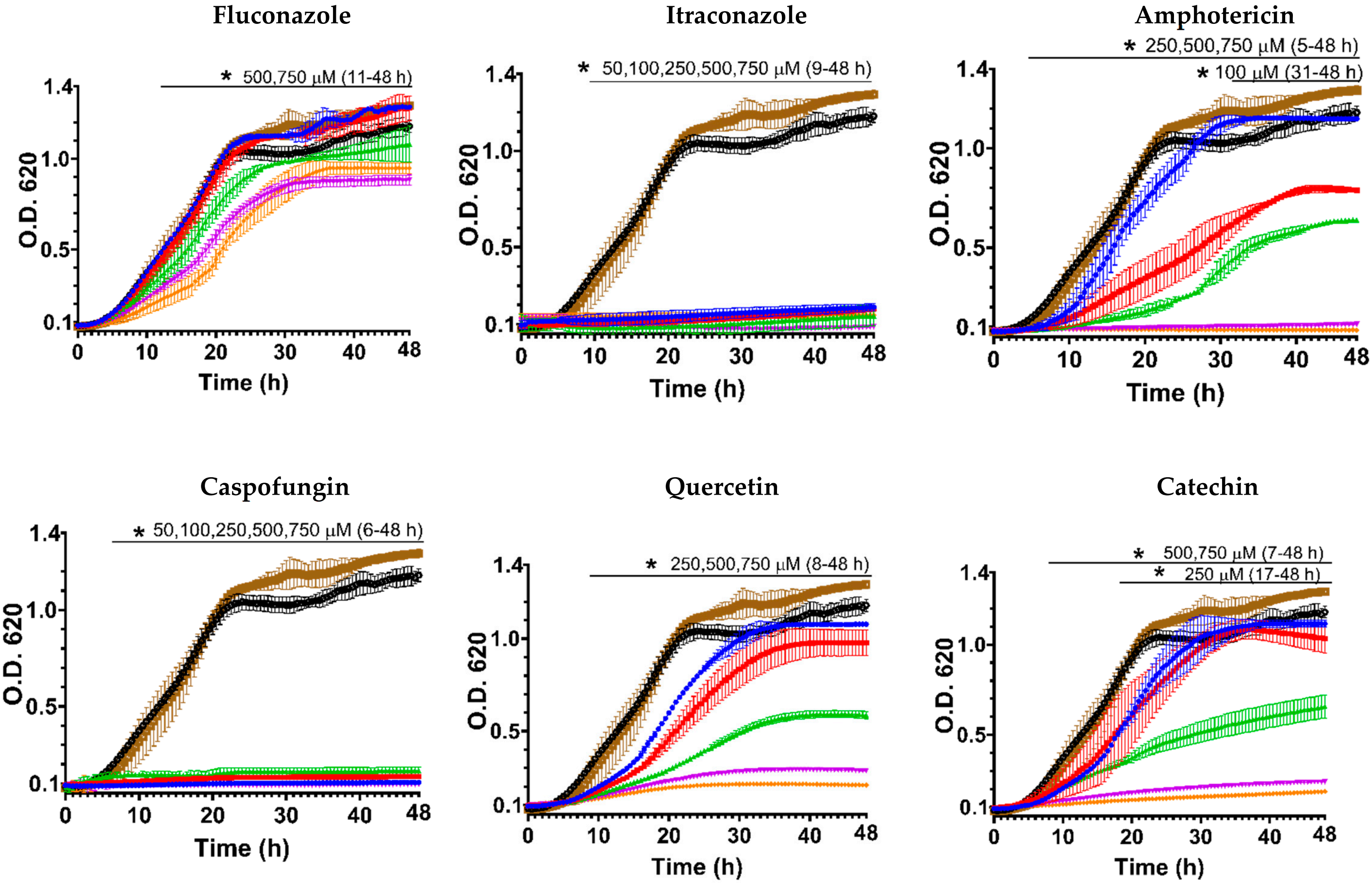

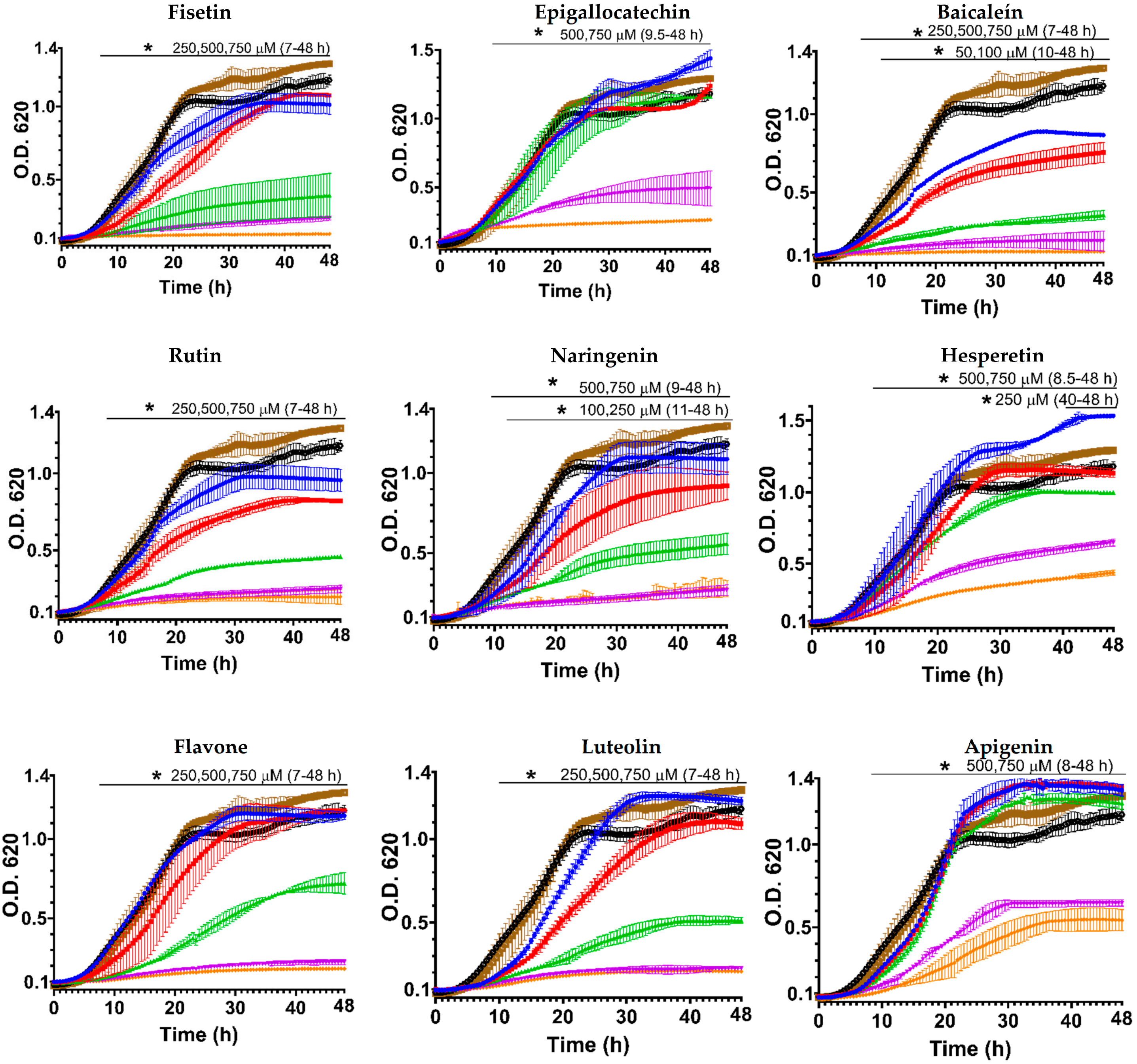

Figure S3. Effect of flavonoids and reference antifungals on the growth of *C. auris* 20-1498. Yeasts were grown in the presence of 50 (blue dots), 100 (red dots), 250 (green dots), 500 (purple dots), and 750 µM (orange dots) as described in the materials and methods section. The controls used were untreated cells (black dotted lines) and cells with DMSO (brown dotted lines). Experiments were performed in triplicate, and the dots represent the mean ± standard error. The horizontal bar indicates the time intervals in which all treatments differed significantly from the untreated (black dots) and DMSO (brown dots) controls (two-way ANOVA, Dunnett's test, * $p < 0.05$, ns = no significant difference).

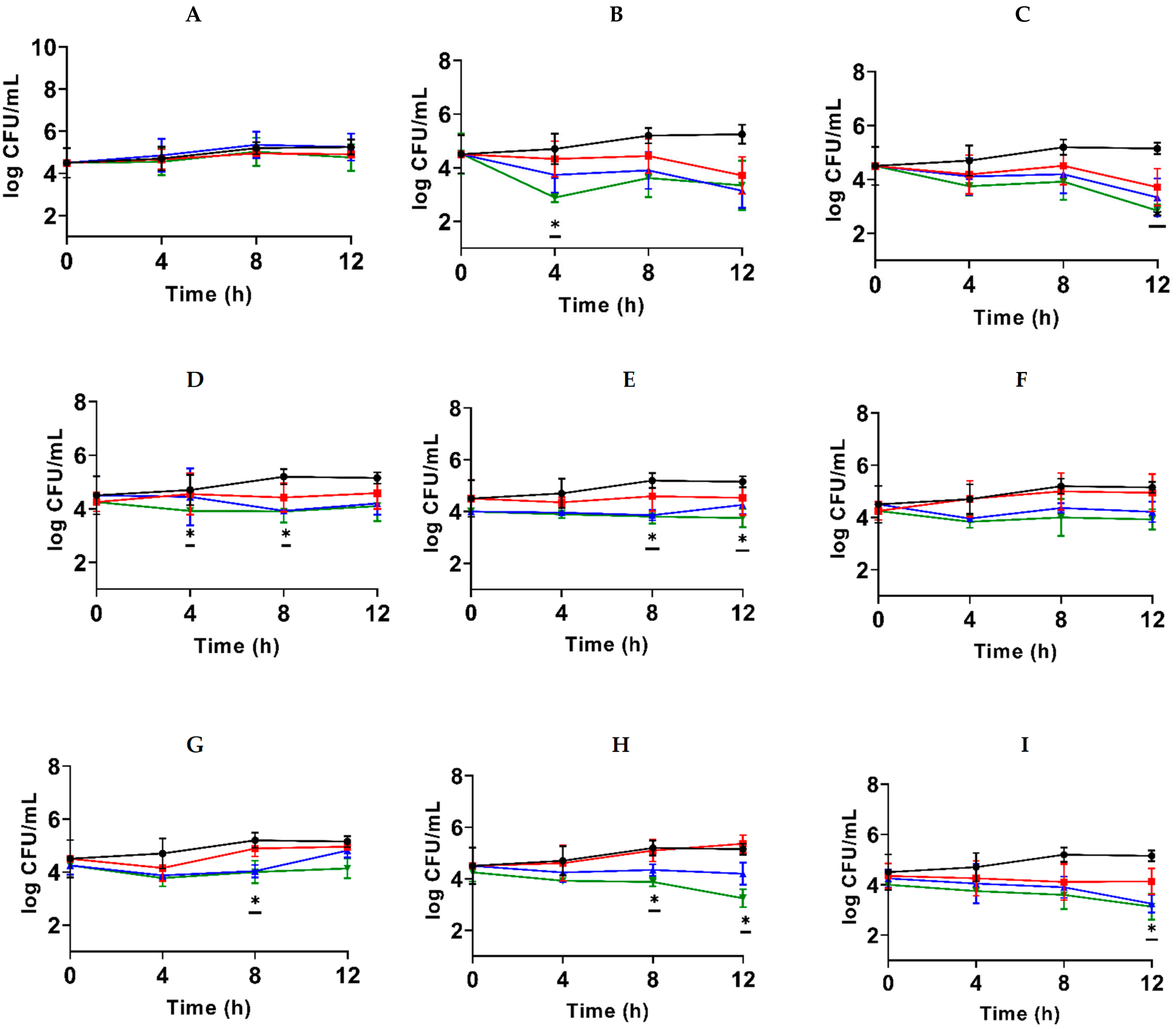

Figure S4. Effects of flavonoids and reference antifungal agents on the viability of *C. auris* CJ97 (clade III). Yeast cultures were incubated at 37 °C in the presence of fluconazole (A), itraconazole (B), amphotericin B (C), catechin (D), rutin (E), naringenin (F), hesperetin (G), luteolin (H), and flavone (I) at concentrations of 250 (red dots), 500 (blue dots), and 750 μM (green dots). Data represents the mean ± standard error of three independent experiments. Asterisks indicate time points at which all treatments differed significantly from the untreated control (black dots), as determined by two-way ANOVA followed by Dunnett's post hoc test (*$p < 0.05$).

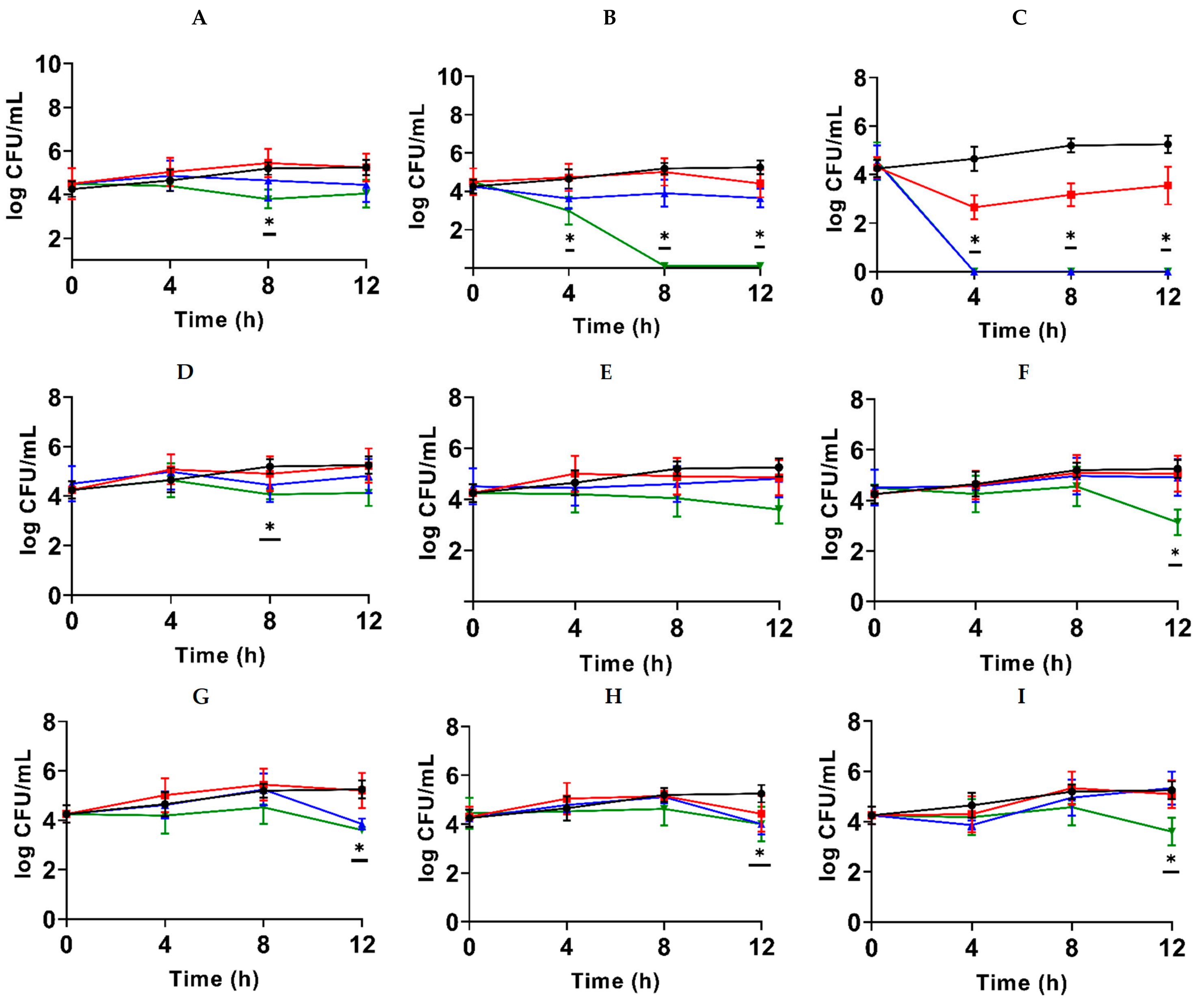

Figure S5. Effects of flavonoids and reference antifungal agents on the viability of *C. auris* 20-1498 (clade IV). Yeast cultures were incubated at 37 °C in the presence of fluconazole (A), itraconazole (B), amphotericin B (C), catechin (D), rutin (E), naringenin (F), hesperetin (G), luteolin (H), and flavone (I) at concentrations of 250 (red dots), 500 (blue dots), and 750 µM (green dots). Viable cell counts were determined at 0, 4, 8, and 12 h. Data represents the mean ± standard error of three independent experiments. Asterisks indicate time points at which all treatments differed significantly from the untreated control (black dots), as determined by two-way ANOVA followed by Dunnett's post hoc test (*p < 0.05).

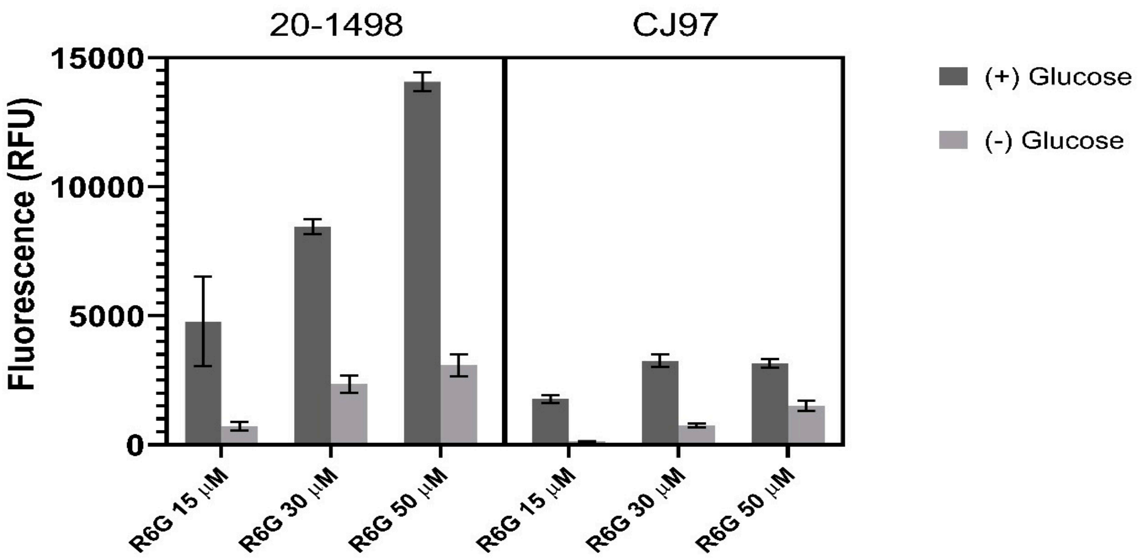

Figure S6. Assessment of ATP-dependent (ABC-type) efflux pump activity in *C. auris* strains 20-1498 and CJ97. Efflux pump behavior was evaluated at increasing concentrations of rhodamine 6G (R6G) in the presence or absence of 8 mM glucose. Glucose supplementation served as an inducer of efflux activity, whereas glucose-free conditions were used as controls for reduced transporter activity.

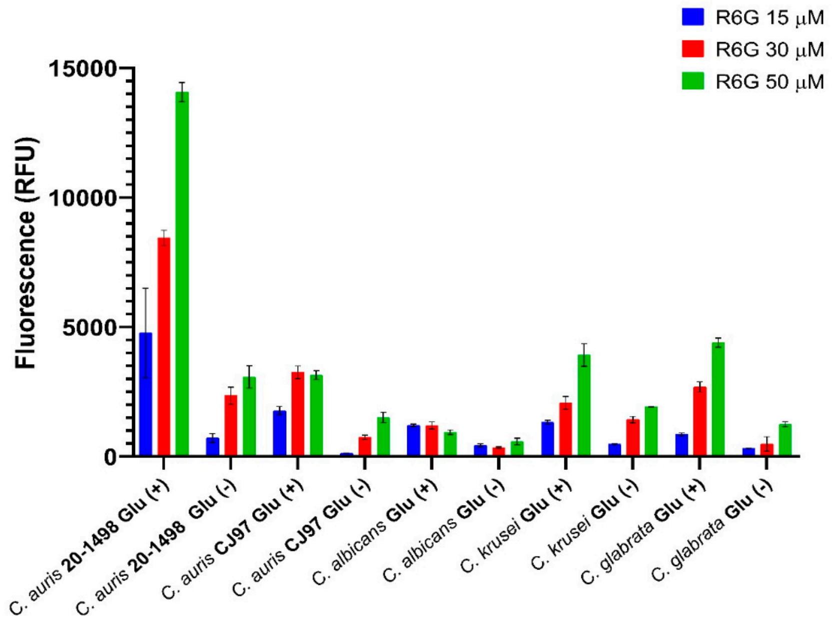

Figure S7. Evaluation of ATP-dependent efflux pump (ABC transporter) activity in *C. auris* strains 20-1498 and CJ97, and in *C. albicans*, *C. krusei*, and *C. glabrata*. Transporter activity was assessed at different concentrations of R6G, with and without 8 mM glucose.

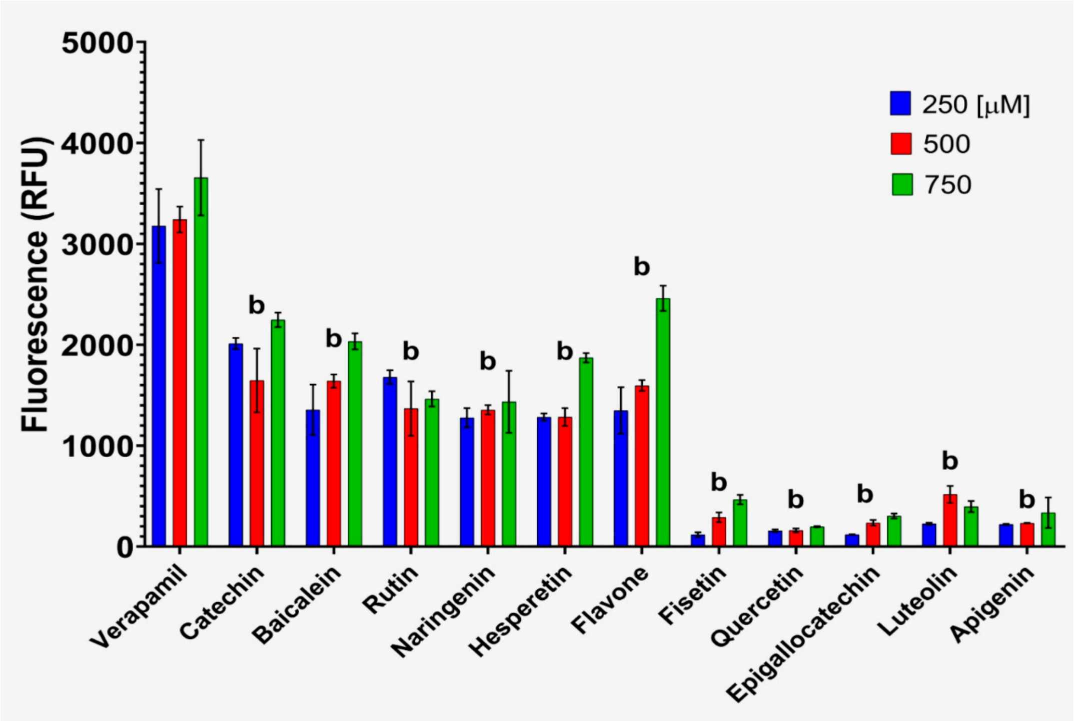

Figure S8. Assessment of ATP-dependent efflux pump (ABC transporter) activity in *C. auris* CJ97. Co-treatment with flavonoids and fluconazole at 250, 500, and 750 μM were used. All assays were conducted using 15 μM R6G in the presence of 8 mM glucose. Distinct letters denote statistically significant differences relative to the verapamil control at corresponding concentrations, as determined by two-way ANOVA followed by Dunnett's post hoc test. Letter **a** indicates $P > 0.05$ versus the verapamil control, whereas letter **b** indicates $P < 0.05$ versus the verapamil control.

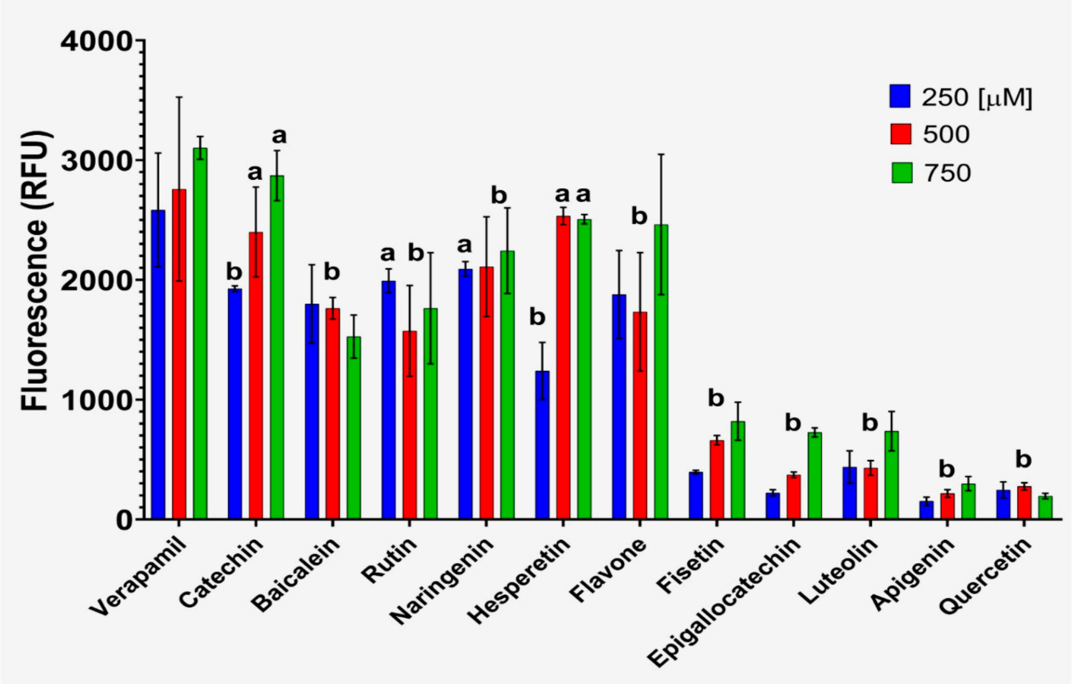

Figure S9. Assessment of ATP-dependent efflux pump (ABC transporter) activity in *C. auris* 20-1498. Co-treatment with flavonoids and fluconazole at 250, 500, and 750 μM were used. All assays were conducted using 15 μM R6G in the presence of 8 mM glucose. Distinct letters denote statistically significant differences relative to the verapamil control at corresponding concentrations, as determined by two-way ANOVA followed by Dunnett's post hoc test. Letter **a** indicates $P > 0.05$ versus the verapamil control, whereas letter **b** indicates $P < 0.05$ versus the verapamil control

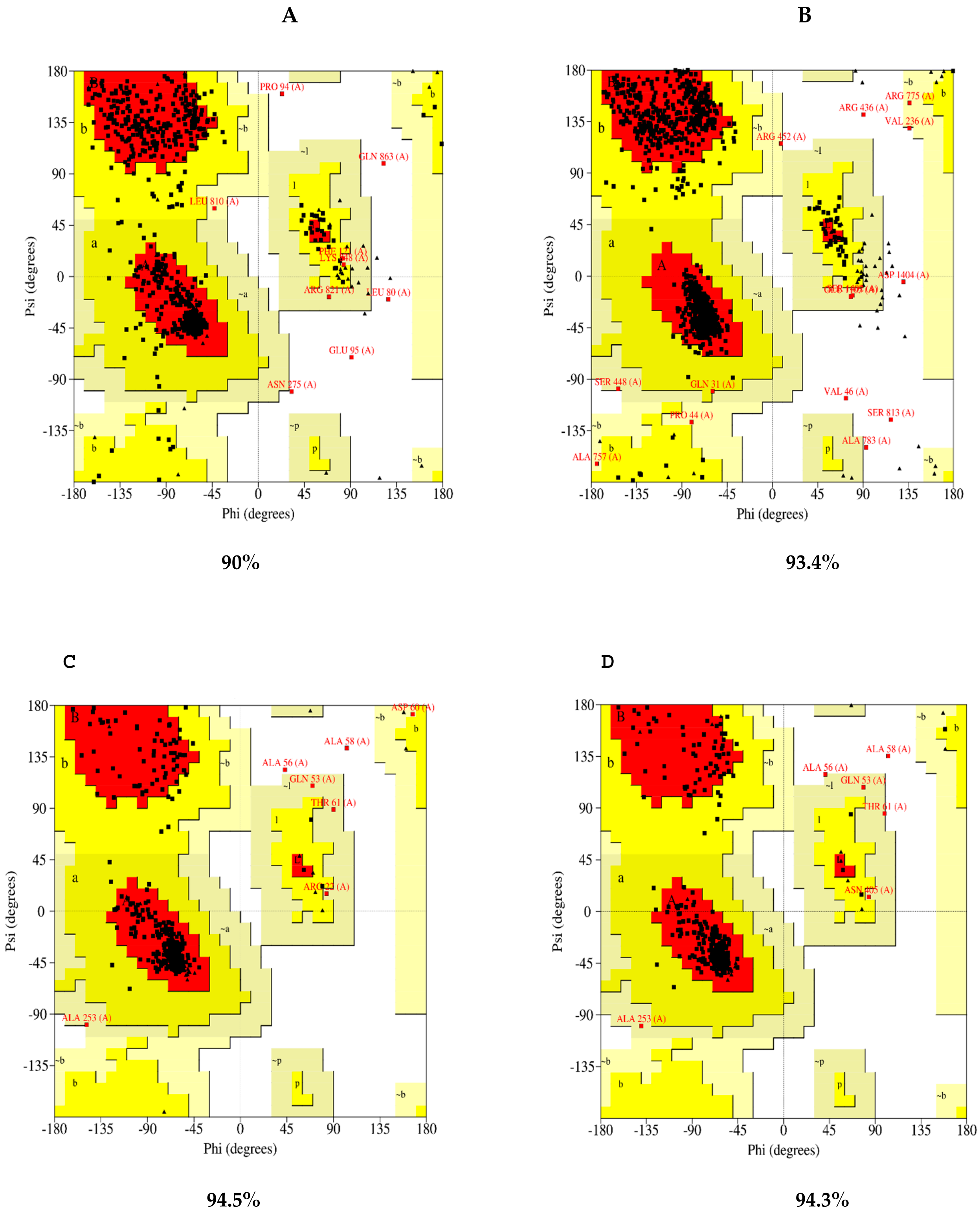

Figure S10. Evaluation of the 3D models of the efflux pumps of *C. auris* from clades III and IV. The Ramachandran plots were generated using the PROCHECK server. For each constructed model, more than 90% of the amino acid residues were located in favored regions, confirming the stereochemical quality of the predicted structures.

Table S1. Minimum inhibitory concentration (MIC) results of flavonoids and reference compounds against *C. auris* (clades III and IV).

**Compounds**	**MIC (µg/mL)**	
	***C. auris* Clade III**	***C. auris* Clade IV**
Fluconazole	>64	>64
Itraconazole	1	1
Caspofungin	0.3	0.5
Amphotericin B	2.5	2
Rutin	0.16	0.16
Hesperetin	0.63	20
Luteolin	1.25	2.5
Naringenin	2.5	0.63
Epigallocatechin	20	40
Baicalein	20	5
Catechin	20	20
Flavone	40	10
Apigenin	20	20
Fisetin	>64	5
Quercetin	>64	>64

Table S2. MIC values converted from μg/mL to micromolar concentrations (μm)

**Compound**	*C. auris* **CJ97** MIC (μg/mL)	*C. auris* **CJ97** MIC (μM)	***C. auris 20-1498*** MIC (μg/mL)	*C. auris* **20-1498** MIC (μM)
Fluconazole	>64	>209	>64	>209
Itraconazole	1	1.42	1	1.42
Caspofungin	0.3	0.27	0.5	0.41
Amphotericin B	2.5	2.71	2	2.16
Rutin	0.16	0.26	0.16	0.26
Hesperetin	0.63	2.08	20	66.2
Luteolin	1.25	4.37	2.5	8.73
Naringenin	2.5	9.18	0.63	2.31
Epigalocatechin	20	65.3	40	130.6
Baicalein	20	74	5	18.5
Catechin	20	68.9	20	68.9
Flavone	40	180	10	44.98
Apigenin	20	74	20	73.98
Fisetin	>64	>224	5	17.46
Quercetin	>64	>212	>64	>211.70

Table S3. Tested concentrations expressed as multiples of the MIC (×MIC) in *C. auris* CJ97

**Compound**	**MIC (µM)**	**50 µM (×MIC)**	**100 µM (×MIC)**	**250 µM (×MIC)**	**500 µM (×MIC)**	**750 µM (×MIC)**
Fluconazole	> value	> or sub-MIC	> or sub-MIC	> or sub-MIC	> or sub-MIC	> or sub-MIC
Itraconazole	1.42	35.21	70.42	176.06	352.11	528.17
Caspofungin	0.27	185.19	370.37	925.93	1851.85	2777.78
Amphotericin B	2.71	18.45	36.90	92.25	184.50	276.75
Rutin	0.26	192.31	384.62	961.54	1923.08	2884.62
Hesperetin	2.08	24.04	48.08	120.19	240.38	360.58
Luteolin	4.37	11.44	22.88	57.21	114.42	171.62
Naringenin	9.18	5.45	10.89	27.23	54.47	81.70
Epigalocatechin	65.30	0.77	1.53	3.83	7.66	11.49
Baicalein	74.00	0.68	1.35	3.38	6.76	10.14
Catechin	68.90	0.73	1.45	3.63	7.26	10.89
Flavone	180.00	0.28	0.56	1.39	2.78	4.17
Apigenin	74.00	0.68	1.35	3.38	6.76	10.14
Fisetin	> value	> or sub-MIC	> or sub-MIC	> or sub-MIC	> or sub-MIC	> or sub-MIC
Quercetin	> value	> or sub-MIC	> or sub-MIC	> or sub-MIC	> or sub-MIC	> or sub-MIC

Concentrations were normalized and expressed as multiples of the MIC (×MIC). However, for compounds with MIC values reported as greater than the highest concentration tested (e.g., > 200 µM), precise ×MIC values could not be determined; consequently, these treatments were classified as "> or sub-MIC" exposures.

Table S4. Tested concentrations expressed as multiples of the MIC (×MIC) in *C. auris* 201498

**Compound**	**MIC (µM)**	**50 µM (×MIC)**	**100 µM (×MIC)**	**250 µM (×MIC)**	**500 µM (×MIC)**	**750 µM (×MIC)**
Fluconazole	> value	> or sub-MIC	> or sub-MIC	> or sub-MIC	> or sub-MIC	> or sub-MIC
Itraconazole	1.42	35.21	70.42	176.06	352.11	528.17
Caspofungin	0.41	121.95	243.9	609.76	1219.51	1829.27
Amphotericin B	2.16	23.15	46.3	115.74	231.48	347.22
Rutin	0.26	192.31	384.62	961.54	1923.08	2884.62
Hesperetin	66.2	0.76	1.51	3.78	7.55	11.33
Luteolin	8.73	5.73	11.45	28.64	57.27	85.91
Naringenin	2.31	21.65	43.29	108.23	216.45	324.68
Epigalocatechin	130.6	0.38	0.77	1.91	3.83	5.74
Baicalein	18.5	2.7	5.41	13.51	27.03	40.54
Catechin	68.9	0.73	1.45	3.63	7.26	10.89
Flavone	44.98	1.11	2.22	5.56	11.12	16.67
Apigenin	73.98	0.68	1.35	3.38	6.76	10.14
Fisetin	17.46	2.86	5.73	14.32	28.64	42.96
Quercetin	> value	> or sub-MIC	> or sub-MIC	> or sub-MIC	> or sub-MIC	> or sub-MIC

Concentrations were normalized and expressed as multiples of the MIC (×MIC). However, for compounds with MIC values reported as greater than the highest concentration tested (e.g., > 200 µM), precise ×MIC values could not be determined; consequently, these treatments were classified as "> or sub-MIC" exposures.
